# Observations on Recent Depressions of the Skull, Unattended with Symptoms

**Published:** 1805-08-01

**Authors:** G. P. Dawson

**Affiliations:** Member of the Royal College of Surgeons in London


### Observations on recent Depressions of the Skull, unattended with Symptoms. *By* G. P. Dawson, *Member of the Royal College of Surgeons in London.*

As no less than four cases of fractured skull, with depression, have made their appearance in the course of the the 13th volume of the Medical and Physical Journal, and as one of these cases seems to inculcate a different practice from that of the other three, it may perhaps be pardonable to offer a few remarks on this species of injury. This consideration is the more necessary, as from the various opinions of authors, and the diametrically opposite methods of treatment proposed by some of the first practitioners in this country, a young surgeon must necessarily feel at a loss to decide on that particular practice he ought to pursue. He may respect all these surgeons, but he cannot believe them all to be right; and while his ideas on this subject are 'wavering and doubtful,' it is hardly to be expected he can act with that promptness and decision which cases of imminent danger most unquestionably demand. It is the conviction of this, and the wish to remedy it, which has given origin to this paper. I have not the vanity to pretend to teach, I only wish to stimulate the industry of others. I shall confine my observations under three distinct heads. First, I shall state the treatment recommended by three very celebrated surgeons, men of acknowledged eminence, whose opinions must give weight to any practice, each of which differ considerably. Secondly, I shall make a few observations on the four cases of depression, published in this Journal, by Messrs. Snow, Pitt, and Goddard. And, thirdly, I shall combine these detached cases with the practice I have seen, heard, and read of; and having commented on the treatment of these surgeons, draw my conclusions accordingly: not, as I have already said, to instruct, but to offer food for discussion, and induce so interesting a subject to be resumed by an abler pen.

In the prosecution of this inquiry it will be superfluous to mention names, or to speak of the practice, in depression, with symptoms; here, there neither is, nor indeed can be, diversity of opinion: all must concur, in one general voice, that the use of the trephine is indispensable. But we are to consider how far the use of that instrument is necessary in cases of depression, unattended with symptoms. To assist us in this consideration then, we are to hear what three famous surgeons say. The first surgeon tells us, in recent cases of depression unattended with bad symptoms, the operation should be performed; that this practice is vindicable on two accounts; first, though there are instances of recovery in such cases, yet when the injury is neglected, and the patient happens to live freely, it is no unusual thing to see inflammation supervene some time after the accident; secondly, long pressure on the brain is apt to derange the functions of this organ.

So much for the first surgeon; and now for the directions of the second, who informs us, that in cases of depression without symptoms, and an external wound, there is no occasion to operate; but should there be depression with a wound leading down to it, then the trephine should be applied.

Let us now hear what the third surgeon says. "Whenever the patient retains his senses perfectly, I should think it improper to trephine him, unless symptoms arose that indicated the necessity of it. The cases which I have related will, I believe, justify me in entertaining this opinion, and in thinking that the contrary practice is now carried by some surgeons to a prejudical extreme."

Here is variation of opinion with a vengeance! According to the first gentleman, we are always to operate for depression; to the second, only with a wound leading downdown to it; to the third, only when symptoms arise to indicate its necessity. How then is a young surgeon to act? By the rules of teachers and authors? No! For, do they not essentially differ? In the hour of danger, then, when every heart beats high with expectation, and friends and attendants look up with an anxious eye to the surgeon for consolation and relief, he, with a mind tortured "'twixt hope and fear," pursues a practice, applauded by some, condemned by others, each of equal celebrity, and each deserving of implicit credit! If these inconveniences could be obviated, and a determinate plan of treatment resolved on, how great the advantage and utility to the profession in general!

I now enter into the second part of my design, or that of making a few comments on the four cases of depression published in the thirteenth volume of this Journal. I shall reverse their order, by first speaking of Mr. Goddard's, then of Mr. Snow's, and lastly of Mr. Pitt's. The communication given us by Mr. Goddard is a valuable one, inasmuch as it gives us eight successful cases of depression without the operation. It would however have been more satisfactory, for the purpose of assisting us in the consideration of the plan of treatment advised by the second surgeon, had he informed us whether the other six cases alluded to, were accompanied with wounds or not. The other two recent cases detailed are highly instructive, and replete with practical facts, as they demonstrate to us, beyond a doubt, that patients labouring under depression of the skull, *with a wound, and even with the existence of symptoms*, may recover without the application of the trephine.

The first case* is that of a farmer, aged twenty-five, which Mr. G. thus relates: "and depressed a part of the frontal bone, nearly the size of a tea spoon bowl; he was stunned and beat down, but after a time got home. Next morning, when I was called, I found symptoms of depression, dilated pupil, great pain in the head, sickness and vomiting, slow pulse, and great loss of strength. The wound was about an inch long, and the pericranium was detached from the bone, so that the depression was plainly to be seen. As the case did not appear urgent, I determined to try the effect of bleeding, copious purgings, with a low regimen, and a dark room, dressing the wound lightly, and was much pleased to find every appearance go

L 2 on

* Medical and Physical Journal, No. 76, p. 526.

on favourably. The man was able to go out in about three weeks, the sore quite healed, and has remained well ever since."

Mr. G. says, "as the case did not appear urgent." Here I must differ in opinion; I think the case was urgent, and might well have justified the operation. I should have operated, and I believe one-tenth part of the profession would have done the same. Yet the event proved he was right; and as such, I think he deserves much credit.

The second case, is that of a boy, aged fourteen, stated as follows: "A blow on the parietal bone, about an inch above the ear; a portion of scalp was detached, and the fracture and depression were more extensive than in the first, and so also were the symptoms; however, the success of former cases induced me to try the same plan, of bleeding and a low regimen, and uniting the scalp by the first intention, which could not be done in the former case, as the parts were bruised and burnt by the powder; and am happy in saying every thing went on to my wish; in three weeks the boy went home to his friends, and has continued well ever since." Here is another case where most surgeons would have operated, yet the consequent success, and forbearance of the operation, are highly creditable to the attending surgeon.

I now come to the third case of depression, transmitted to us by Mr. Snow.* "A soldier, without the least provocation, struck his comrade, who was asleep, and lying upon a form rather in liquor, with a flat piece of iron, about two feet and a half in length, and about six pounds weight, upon the parietal bone, two inches and a half in a perpendicular direction above the extreme edge of the external ear; the scalp was divided nearly three inches longitudinally, and the fracture continued the whole length of the wound, together with the depression of the upper edge of the bone, so that the inferior edge could be very distinctly felt, as well as a small piece of the upper table of the skull, loosely detached, but adhering firmly to the scalp. The blow had divided the continuation of the temporal artery; and as it was some length of time before his situation was discovered, when I went down to him, I found he had lost full two pints of blood. After taking up the artery, and finding the man perfectly sensible, I determined not to use the trephine until the symptoms warranted

* Medical and Physical Journal, No. 74, p. 295.

ranted it; and closing the wound by two stitches, left him for the night. In the morning (being December 4) I was much pleased to see him entirely free from every appearance of coma, delirium, or even fever; I gave him a saline purge, which operated three or four times copiously. In the evening I repeated my visit; I thought his pulse a little too full, although he did not complain of any pain. I took from his arm sixteen ounces of blood, and he expressed himself 'lighter' for its loss. I kept him on very low diet, and continued seeing him three or four times during the day; no alarming symptom occurred. On the fifth day from the accident his pulse felt stronger than I wished it, and I was very particular in my inquiries respecting his feelings, but he assured me he felt as well as usual; however, I considered it better, if possible, to prevent the approach of inflammation, and I bled him to sixteen ounces, as before, and no return of fulness existed afterwards. During the whole time of his illness, his appetite continued very good, and his bowels regular; and as no fever, or any other bad symptom was induced, I conceived medicines to be useless. On the 21st of December, I reported him convalescent."

This case, like those of Mr. Goddard's, is extremely valuable. It were an unnecessary task to comment much on this case; let it suffice to say that it affords a convincing proof, in addition to many others, that though depression may be attended with a wound, yet still the application of the trephine may be safely dispensed with. Mr. S. says, "The blow had divided the continuation of the temporal artery; and, as it was some length of time before his situation was discovered, when I went down to him I found he had lost full two pints of blood."——"In the morning I was much pleased to see him entirely free from every appearance of coma, delirium, or even fever." Was not this man's favourable situation in the morning, chiefly owing to the liberal quantity of arterial blood he had lost from the division of the temporal artery? And does not this strongly indicate the utility and advantage which are to be derived from free and repeated bleedings in similar cases?

The fourth and last case, recited by Mr. Pitt, now presents itself to my notice. The following is Mr. P's account of the case.* "On clearing away the blood and

 shaving

* Medical and Physical Journal, No. 75, p. 445.

shaving his head, I perceived a large wound on the right side and towards the vertex, near the posterior and superior angle of the parietal bone, part of which was laid bare; it extended forward about four inches in length, parallel with the sagittal suture. As the *mental faculties* of the boy were *not at all impaired,* I did not at first conceive that any fracture had taken place, more particularly as the bone appeared perfectly smooth and without fissure when laid bare; but on lifting up the scalp and examining further, I found I was mistaken in my conjecture. I immediately dilated the wound, and discovered that the parietal bone *was fractured and depressed,* at least three inches in length, and three-quarters of an inch in breadth near the posterior and superior angle, and passing forwards nearly in the direction of the wound. I immediately applied the trephine, and raised the depressed part; no effusion had taken place; the scalp was laid down, dressed with mild digestive, and the dressings secured by a bandage and a cotton night cap. The patient (I was informed by the officer who brought him) appeared rather convulsed as he came in the boat; but when brought on board the ship he had no symptoms of them; on the contrary, he was perfectly sensible, and answered any questions that were put to him, and continued so during the whole time of the operation. During the first night after the operation he appeared rather delirious for a short time once or twice, and had some slight febrile symptoms, and the four or five succeeding nights had a slight return of fever without delirium; after which he had no complaint, except a slight head-ach now and then, until the completion of the cure."

This case is less valuable than the preceding ones, as it is only a proof of the operation succeeding in a case of depression, without symptoms. After having related this case, Mr. Pitt goes on to say, "In the preceding case, not one of the symptoms generally enumerated, viz. stupor, loss of sense and voluntary motion, dilated pupil, &c. were present, although the depressed part of the bone was pressing firmly on the dura mater; and had there been no external wound, it might have been difficult to have ascertained the existence of a fracture; in which case the patient would have been freely bled, had purgative medicines given him, and he would have been supposed to be doing well, until, in a short time, all the alarming symptoms would have shewn themselves; violent inflammation would in all probability have taken place in the membranes branes of the brain, which might have terminated fatally." All these, to use the language of the ingenious Dr. Kinglake, are gratuitous conclusions, controverted by observation, witness the successful cases of depression related by Mr. Abernethy,* in his Surgical Essays, and others, where no operation was performed. Mr. P. asks, "If it was not to be presumed they (the symptoms in the boy's case) would shortly have taken place, if the piece of bone in the compression had not been elevated?" Though it is very possible, alarming symptoms might have come on, the probability is they might not; but let Mr. Goddard's cases answer this question,—successful, without the operation, though evidently worse than this case, as the pericranium was not only detached from the bone, but *bad symptoms actually existing*.

Before I go further, let me assure the ingenious Reporter, that in thus freely commenting on his case, and remarks, I am actuated by no disrepect, or wish to cavil at his treatment; far, very far from it: nay, I will go so far as to say, that in the case in question, where "the nature of the climate is considered, where, from the excessive heat, determination to the head is easily induced," the operation was warrantable, and perhaps the most prudent practice that could be adopted. It now remains for me to proceed to the third consideration, or that of commenting on the practice of the surgeons, whose opinions I have previously stated; and by comparing detached cases and observations, draw a conclusion respecting the propriety of operating in cases of depression without symptoms. The first surgeon advises "the operation in recent depressions, for two reasons; the first, because inflammation may supervene; the second, long pressure is apt to derange the functions of the brain."

Let us examine these opinions singly. First, or "inflammation may supervene." This circumstance, though it certainly may occur, is yet comparatively rare; this is proved by numerous and well attested cases of success published by Mr. Abernethy, and other eminent surgeons. The fear of inflammation supervening therefore, some time after the injury is received, in my opinion, cannot justify the operation; it is using the trephine as a prophylactic: and to use a strange, but certainly applicable simile, it is something like putting a man under a course of mercury *post*

* Abernethy's Surgical Essays, vol. iii.

*coitu* with a Cyprian goddess, under the supposition of his having received a syphilitic infection! And now for the second, or "long pressure is apt to derange the functions of the brain." This, though its possibility cannot be denied, an instance of which I have myself seen, is yet best answered by the observations of several excellent surgeons, particularly by the ingenious Mr. John Bell,* of Edinburgh; and in addition to this, and other authority, I may state the information of a friend of mine, who during his residence in Ireland, in a military capacity, saw several cases of depression of the skull, where no operation was employed; and though a considerable time had then elapsed, the subjects of these cases remained perfectly well.

Still further to strengthen the remarks I have presumed to make on these opinions, I might adduce what has been said on this subject by the justly celebrated Mr. Abernethy. But as his interesting Essays are universally read, this appears to be unnecessary.

The second surgeon tells us, "Operate only for depression with a wound leading down to it." Neither can this, in my mind, justify the use of the trephine; for though bad consequences may occur from neglecting to operate, yet the cases of Mr. Abernethy and other surgeons, shew that this is by no means to be frequently expected; or, if it is, why did not danger ensue from the cases of Mr. Goddard; cases where the pericranium was detached from the bone, and *bad symptoms actually existing?* or in the case, published in this Journal by Mr. Snow?

The third surgeon says, "Whenever the patient retains his senses perfectly, I should think it improper to trephine him, unless symptoms arose that indicated the necessity of it. The cases which I have related will, I believe, justify me in entertaining this opinion, and in thinking that the contrary practice is now carried by some surgeons to a prejudicial extreme." What comments am I to offer on this practice? None! I believe none to be required. I have promised in a former part of this paper, to draw a conclusion respecting the propriety of operating in cases of depression without symptoms; but that modesty and caution, which I hope, in my writings, I shall never violate, withholds my pen, and whispers me to forbear. The opinion I have ventured to form, in my own mind, is, I flatter

* Discourses on Wounds, by John Bell, vol. ii.

flatter myself, from what I have already written, sufficiently evident. I meant to say,—but enough of this, I will not presume to decide, I leave the task to abler and more experienced surgeons.

" Let those teach others who themselves excel,
" And censure freely who have written well."

*Sunderland, June* 15, 1805.

